# Witnessing Interparental Violence and Adolescents’ Non-Suicidal Self-Injury: Differential Mediating Roles of Presence of Meaning and Search for Meaning

**DOI:** 10.3390/bs16101866

**Published:** 2026-10-10

**Authors:** Xiujuan Wu, Keyao Wang, Yaru Shi, Xun Zhou

**Affiliations:** College of Education, Huaibei Normal University, Huaibei 235000, China; 12412050505@chnu.edu.cn (K.W.); 12412050361@chnu.edu.cn (Y.S.); zhoux@chnu.edu.cn (X.Z.)

**Keywords:** adolescents, non-suicidal self-injury, the presence of meaning, the search for meaning, witnessing interparental violence

## Abstract

Most prior research on adolescents’ exposure to interparental violence has focused on externalizing outcomes such as aggressive behavior, whereas relatively little attention has been given to NSSI. This study aimed to examine the association between witnessing interparental violence and adolescents’ non-suicidal self-injury (NSSI) by exploring the mediating roles of presence of meaning and search for meaning and examining gender as a potential moderator in an exploratory manner. Data were collected from 1673 adolescents (*M*age at Time 1 = 13.14 years, *SD* = 0.78; 48.12% girls) recruited from three public junior high schools in China. Participants completed measures of witnessing interparental violence, meaning in life, and non-suicidal self-injury (NSSI) across three waves at six-month intervals. Results indicated that witnessing mother-to-father violence (WMFV) at Time 1 had a significant total effect on NSSI at Time 3, whereas witnessing father-to-mother violence (WFFV) at Time 1 had a marginally significant total effect, after controlling for gender, age, socioeconomic status (SES), and baseline NSSI. After presence of meaning (ML-P) and search for meaning (ML-S) were included as mediators, only ML-P, and not ML-S, significantly mediated these associations. The exploratory analysis of gender moderation did not reveal significant gender differences in these associations. These findings suggest that ML-P may be one pathway linking witnessing interparental violence to NSSI among adolescents. Given the observational design, causal inferences cannot be drawn. Nevertheless, the results are consistent with the view that preventing interparental violence and supporting adolescents’ sense of meaning may be relevant for reducing NSSI risk, and these possibilities warrant further investigation.

## 1. Introduction

The detrimental effects of witnessing interparental violence on adolescents’ psychological and physical health have been extensively documented (e.g., Haj-Yahia & Basal, 2024; T. Lawrence, 2022). However, previous research has primarily focused on externalizing outcomes such as aggressive behavior, with relatively little attention paid to NSSI. Non-suicidal self-injury (NSSI) refers to the deliberate destruction of one’s own body tissue without suicidal intent (Nock & Favazza, 2009) and is one of the most common maladaptive behaviors in adolescents (Daukantaitė et al., 2019), with cutting or carving the skin using a sharp object being the most frequent method (You et al., 2013). Prior studies have also shown that NSSI is often associated with more severe psychopathology, including depression, anxiety, and, in some cases, suicidal behavior (Glenn & Klonsky, 2013; Muehlenkamp et al., 2023; Selby et al., 2012). Given the limited research on the link between witnessing interparental violence and adolescents’ NSSI, the first aim of the present study was to examine the longitudinal association between witnessing interparental violence and NSSI among adolescents.

It is equally important to understand the mechanisms through which witnessing interparental violence is associated with adolescents’ NSSI. The stress process model (Pearlin et al., 1981) provides a useful framework for this question. Guided by this model, exposure to stressors depletes individuals’ psychological resources, and the erosion of these resources in turn increases vulnerability to maladaptive outcomes. Meaning in life can be conceptualized as a psychological resource, and its depletion may elevate individuals’ risk of experiencing maladaptive outcomes (King & Hicks, 2021). Accordingly, frequent exposure to interparental violence may be associated with lower levels of meaning in life (Bara et al., 2018; Miranda et al., 2021), and lower meaning in life may in turn be associated with higher NSSI risk. Notably, meaning in life comprises two dimensions: the presence of meaning (ML-P), which reflects the extent to which individuals already experience their lives as meaningful, and the search for meaning (ML-S), which reflects an active effort to understand or enhance meaning (Steger et al., 2006). These two dimensions are related but functionally distinct (King & Hicks, 2021), and examining them separately can clarify whether one or both dimensions are associated with NSSI. Therefore, the second aim of the present study was to examine whether the presence of meaning (ML-P) and the search for meaning (ML-S) would mediate the relationship between witnessing interparental violence and adolescents’ NSSI. Additionally, gender may moderate the association between witnessing interparental violence and adolescents’ NSSI, although findings on gender differences in this area are mixed. Given the absence of consistent evidence supporting a directional hypothesis, the third aim of this study was to examine gender as a potential moderator in an exploratory manner.

### 1.1. Witnessing Interparental Violence and Adolescents’ NSSI

As noted earlier, NSSI is conceptually distinct from suicidal ideation and attempts (Nock & Favazza, 2009). Evidence specific to NSSI is therefore needed to understand its antecedents. NSSI is commonly conceptualized within functional models, which posit that it serves to regulate or escape from aversive internal experiences such as negative affect (Nock & Prinstein, 2004; Chapman et al., 2006). From this perspective, identifying the stressors and psychological resources that shape adolescents’ capacity to regulate distress is critical for understanding NSSI risk. Exposure to violence between parents may function as a chronic stressor for adolescents (Davies et al., 2011), contributing to dysregulation of the stress response system and increased vulnerability to maladaptive outcomes. Rajhvajn Bulat et al. (2024) found that adolescents with prolonged NSSI reported more adverse childhood experiences, particularly domestic violence (including witnessing violence between parents), than those who stopped. However, their measure captured a broader range of adversities than witnessed interparental violence alone. Similarly, S. Zhang and Wang (2026) found that witnessing family violence, including interparental violence, was significantly associated with NSSI among Chinese high school students. Based on these findings, we hypothesized that witnessing interparental violence would be associated with higher levels of NSSI among adolescents.

### 1.2. Adolescents’ Meaning in Life as the Mediator

To understand why witnessing interparental violence is associated with NSSI, we draw on the stress process model (Pearlin et al., 1981). According to this model, exposure to stressors depletes individuals’ psychological resources, and the erosion of these resources in turn increases vulnerability to maladaptive outcomes. Meaning in life can be conceptualized as one such psychological resource: it helps individuals interpret and integrate stressful experiences and maintain a sense of purpose (King & Hicks, 2021). Prior research has shown that a harmonious and stable family environment supports the development of meaning in life (Shek et al., 2025; Soltani Benavandi et al., 2019), whereas conflicting family atmosphere is associated with lower meaning in life (R. Zhang et al., 2017). R. Zhang et al. (2017) tested meaning in life as a mediator between interparental conflict and suicidal ideation in Chinese adolescents and found a significant indirect effect. However, their study focused on interparental conflict rather than interparental violence, which is a specific and extreme form of conflict, and they examined suicidal ideation rather than NSSI. Building on the above indirect evidence, we expected that witnessing interparental violence would be negatively associated with adolescents’ sense of meaning in life.

Furthermore, evidence specific to NSSI supports a negative association between meaning in life and NSSI. For example, Fan et al. (2024) found a significant negative association between adolescents’ sense of meaning in life and NSSI in a cross-sectional study. Similarly, Sanz-Sendra et al. (2024) reported that meaning in life was associated with NSSI in adolescents and suggested that interventions promoting meaning may help prevent NSSI. Together, the evidence on witnessing interparental violence and meaning in life, and on meaning in life and NSSI, suggests that witnessing interparental violence may be associated with lower meaning in life, which in turn may be associated with higher NSSI. We therefore hypothesized that meaning in life may serve as a mediator of the association between witnessing interparental violence and adolescents’ NSSI.

We further distinguished between the two dimensions of meaning in life because ML-P and ML-S are interrelated yet independent, forming a dynamically evolving relationship through which individuals integrate their perceptions of the self and the world (Steger et al., 2011), and they may function differently as mediators. Consistent with this distinction, King and Hicks (2021) emphasized that the two dimensions may function differently: ML-P reflects an established psychological resource that can protect against maladaptive outcomes, whereas ML-S reflects an active search that may arise when meaning is felt to be insufficient. Although the distinction between ML-P and ML-S is well established, much of the existing evidence has examined meaning in life as a single, overarching construct. The studies that have distinguished between the two dimensions have yielded inconsistent findings: some have found that ML-P, rather than ML-S, protects against psychological problems (e.g., Aliche et al., 2023; Onyekachi et al., 2024), whereas others have reported that both dimensions significantly influence mental health (e.g., Aruta, 2023; Brassai et al., 2012). Given these inconsistent findings, the present study examined ML-P and ML-S as separate mediators. We hypothesized that witnessing interparental violence would be negatively associated with both ML-P and ML-S and that ML-P and ML-S would in turn be negatively associated with NSSI.

### 1.3. Gender Differences

The moderating role of gender in the association between witnessing interparental violence and NSSI remains less examined than the mediating role of other psychological processes. Some studies suggest that the impact of exposure to parental violence on psychosocial outcomes is more pronounced in males than in females (e.g., T. I. Lawrence et al., 2023; Sokar et al., 2023), whereas other studies report no such differences (e.g., Fagan & Wright, 2011; Gao et al., 2025). For example, T. I. Lawrence et al. (2023) found that males exposed to family violence were more likely to report depressive symptoms and substance use. Regarding externalizing behaviors, Sokar et al. (2023) revealed that males who witness interparental physical violence are more likely than females to report physical and psychological intimate partner violence. In contrast, Gao et al. (2025) did not observe significant gender differences in the association between witnessing intimate partner violence and adolescents’ dating violence perpetration. In contrast to the mediation hypotheses, which were directional, we did not specify a directional hypothesis for gender moderation because the literature on gender differences in this area is mixed. Instead, we examined gender as a potential moderator in an exploratory manner.

### 1.4. The Current Study

**Figure 1:**
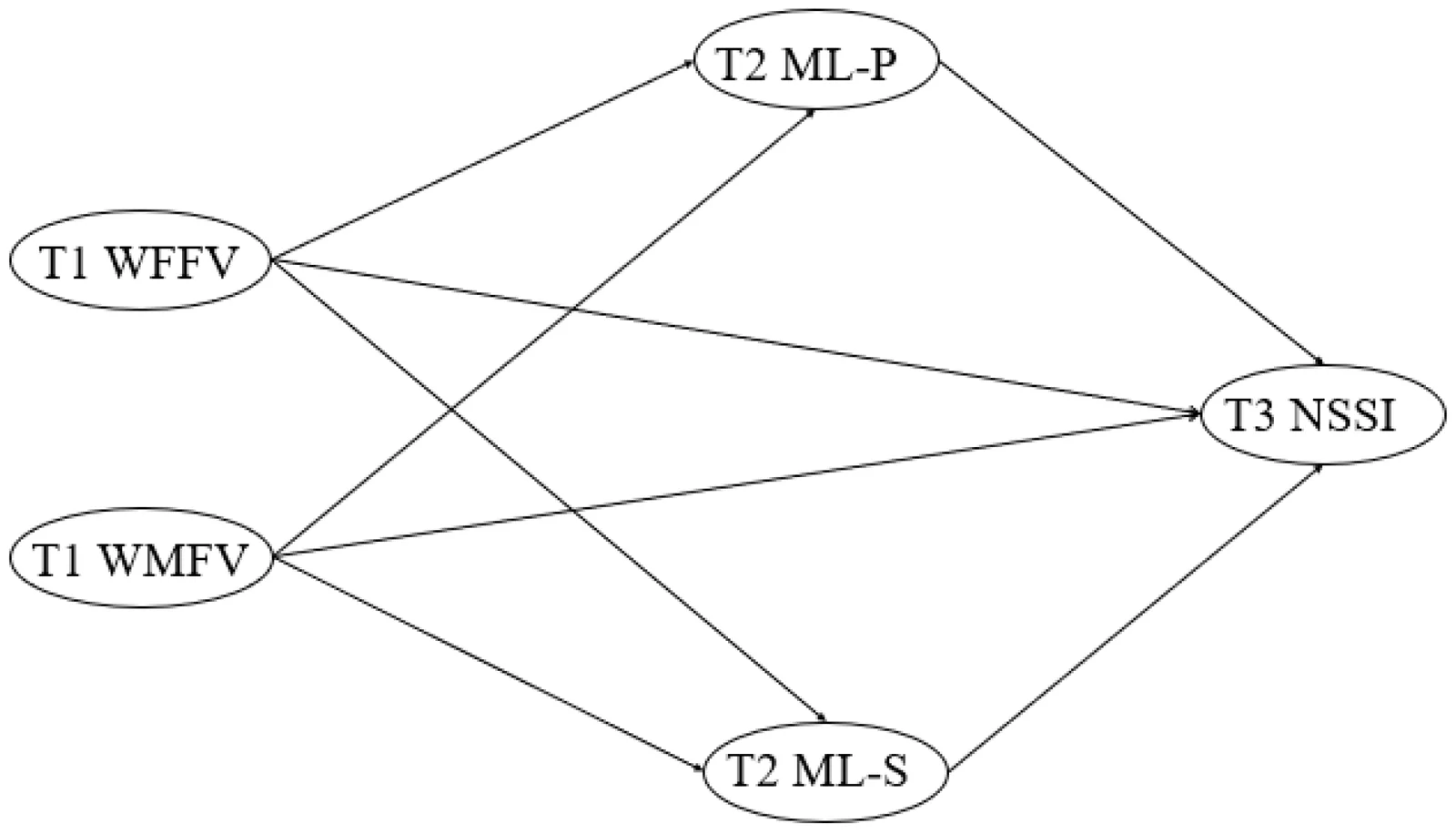
Conceptual Mediation Model. Note. WFFV = witnessing father-to-mother violence, WMFV = witnessing mother-to-father violence, ML-P = presence of meaning, ML-S = search for meaning, NSSI = non-suicidal self-injury.

Drawing on relevant theories and prior empirical research, the present study employed a longitudinal design to examine (see Figure 1) (a) the extent to which witnessing interparental violence is associated with subsequent NSSI among adolescents, controlling for age, gender, SES, and baseline NSSI; (b) whether meaning in life mediates the association between witnessing interparental violence and adolescents’ NSSI; and (c) whether adolescents’ gender moderates the direct and indirect pathways, examined in an exploratory manner.

## 2. Materials and Methods

### 2.1. Participants

Three public junior high schools were selected based on geographic region and willingness to participate, with two schools in Henan Province and one in Shaanxi Province, China. Within each school, students in Grades 7 and 8 were invited to participate, as Grade 9 students were preparing for the high school entrance examination and were therefore not included in the invitation. Only students who lived with both parents were included in the study. A total of 1976 students were invited at Time 1 (March 2025), and 1673 (84.67%) participated. The final sample ranged in age from 12 to 16 years (*M* = 13.14, *SD* = 0.78), and 48.12% were girls. Participants were distributed across the three schools as follows: School 1 (*n* = 385), School 2 (*n* = 707), and School 3 (*n* = 581). Of the original sample, 1543 students (attrition rate = 7.77%) attended the assessment at Time 2 (September 2025), and 1554 students (attrition rate = 7.11%) attended the assessment at Time 3 (March 2026). Of the 1673 participants at T1, 119 (7.11%) participated in only one of the two follow-up waves—that is, some attended T2 but not T3, while others attended T3 but not T2. A further 65 participants (3.89%) did not participate in either follow-up wave and were considered actual dropouts. The attrition was primarily due to students being absent on the day of assessment. Normality tests indicated that T1 WFFV, T1 WMFV, T1 NSSI, and T3 NSSI were somewhat skewed, so we retained only participants who completed all three waves for the main analyses to avoid additional distributional assumptions through missing-data imputation (Dong & Peng, 2013). Attrition bias was evaluated by comparing these 1489 completers with the 184 non-completers using chi-square tests for categorical variables and Mann–Whitney U tests for continuous variables, and no significant differences emerged on the key variables.

In addition, Little’s MCAR test on the full sample (*N* = 1673) was significant (χ^2^(1201) = 2059.18, *p* < 0.001), indicating that wave-level missingness was not completely at random. However, MCAR is a special case of MAR, and rejecting MCAR does not rule out MAR (Schafer & Graham, 2002). In this study, attrition analyses revealed no systematic differences on observed variables, and missingness was primarily due to intermittent participation (119 participants attended only one follow-up wave) and a small number of actual dropouts (65 participants), which is consistent with a plausible MAR mechanism. We retained only participants who completed all three waves for the main analyses (*N* = 1489). Within this sample, a small proportion of item-level data was missing. These missing values were handled using FIML estimation, which utilizes all observed information under the MAR assumption (Enders, 2010; L. K. Muthén & Muthén, 1998–2017).

### 2.2. Measures

#### 2.2.1. Witnessing Interparental Violence (T1)

Witnessing interparental violence was assessed using an adapted version of the eight-item witnessing violence scale developed by Ho and Cheung (2010). The original scale measured the frequency of witnessing violence among family members in general, without specifying the perpetrator–victim relationship. Because existing scales rarely focus specifically on witnessing violence between parents, we adapted the scale by retaining the eight violent behaviors and specifying the perpetrator and victim as the parents in each item. The eight items cover physical violence (e.g., pushing, slapping, twisting arms or grabbing hair/neck, beating up, burning or scalding, and attacking with an object) and threats/property violence (threatening and robbing), and the construct refers to witnessing physical violence and threats between parents.

This yielded two parallel versions—witnessing father-to-mother violence (WFFV) and witnessing mother-to-father violence (WMFV)—each containing eight items. For example, the original item “Have you ever seen somebody being slapped?” was adapted into “Have you ever seen your father being slapped by your mother?” (WFFV) and “Have you ever seen your mother being slapped by your father?” (WMFV). Both versions used the same eight behaviors and the same response options, rated on a 5-point scale ranging from 1 (*never*) to 5 (*daily*). Scores were computed by averaging the eight items, with higher scores reflecting a higher frequency of threatened or actual interparental violence witnessed by the adolescent. The reference period was “ever.” As threatening and robbing were each represented by a single item, they could not form separate factors. We therefore compared a one-dimension model (all eight items on a single factor) with a two-dimension model (physical violence items vs. threat/property violence items) separately for WFFV and WMFV. Confirmatory factor analyses supported the unidimensional structure of both subscales, as a two-dimension model (physical violence vs. threat/property violence) did not fit better than a one-dimension model for either WFFV or WMFV. In addition, to examine whether the two versions were empirically distinguishable, we compared a two-factor model (eight WFFV items on one factor, eight WMFV items on the other, factors allowed to correlate) with a one-factor model (all 16 items on a single factor). Confirmatory factor analyses supported the two constructs as distinguishable. Full model comparison results are reported in Appendix A (Table A1 and Table A2). The Cronbach’s alpha values for the WFFV and WMFV scales were 0.96 and 0.91, respectively.

#### 2.2.2. Meaning in Life (T2)

Meaning in life was assessed using Meaning in Life Questionnaire (MLQ; Steger et al., 2006), which consists of 10 items, with five items on each of the following two dimensions: presence of meaning (e.g., “My life has a clear sense of purpose.”) and search for meaning (e.g., “I am seeking a purpose or mission for my life.”). All items were rated on a 7-point scale ranging from 1 (*absolutely untrue*) to 7 (*absolutely true*). One item on the ML-P subscale was reverse-scored before averaging. For the ML-S subscale, no reverse scoring was required, and the five items were averaged directly. Higher scores on both subscales denote higher levels of the corresponding construct. The MLQ has shown acceptable reliability and validity in assessing meaning in life among Chinese adolescents (e.g., Li & Liu, 2023; Ye et al., 2025). In this study, the Cronbach’s alpha values for the ML-P and ML-S scales were 0.83 and 0.92, respectively.

#### 2.2.3. Non-Suicidal Self-Injury (T1 and T3)

The Chinese version of the Non-Suicidal Self-Injury Scale (You et al., 2013) was used to measure the frequency of adolescents’ NSSI in the past 6 months. This scale consists of seven items (e.g., “deliberate occurrence of behaviors such as self-cutting, biting, or scratching skin”) rated on a 4-point scale ranging from 1 (*never*) to 4 (*six times or more*). Responses across the seven items were averaged, with higher scores denoting a higher frequency of NSSI. This scale has been widely used with Chinese adolescents and has shown acceptable reliability and validity (e.g., Huang et al., 2022; Yu et al., 2020). The Cronbach’s alpha values were 0.89 and 0.93 at T1 and T3, respectively.

#### 2.2.4. Covariates

Prior research has suggested that adolescents’ age, gender, and family SES are associated with NSSI (Gu et al., 2024; J. Tang et al., 2018). Thus, we included these variables as covariates in our subsequent statistical analyses. Age was obtained at the first time point. In this study, gender referred to biological sex as recorded in the school registers. Gender was coded as a dichotomous variable (0 = male, 1 = female). Consistent with the approach adopted in existing literature (e.g., Liu et al., 2024; Wang, 2017), SES is operationalized as the mean of the standardized scores of four indicators: paternal educational attainment, maternal educational attainment, paternal monthly income, and maternal monthly income. Parental educational attainment was coded as follows: 1 = junior high school or below, 2 = high school diploma, 3 = bachelor’s degree, 4 = master degree or above. Most participants’ parents had an educational attainment of high school or below (78.54% of fathers and 78.66% of mothers had a high school degree or lower). The monthly salary (abbreviated as MS hereafter) of respondents’ parents is quantified in Renminbi (RMB) and coded as follows: 1 = MS ≤ 2000, 2 = 2000 < MS ≤ 3000, 3 = 3000 < MS ≤ 4000, 4 = 4000 < MS ≤ 5000, 5 = MS ≥ 5000. Regarding the average monthly income of parents, 75.07% of fathers and 55.17% of mothers have an average monthly income exceeding 3000 RMB.

### 2.3. Procedure

All data collection procedures were approved by the Research Ethics Committee (Approval No. HBNU-2025030901). Well-trained psychology postgraduates conducted the survey after informed consent was obtained from parents and students. Data collection was organized in the respondents’ classes. Participants were informed that their participation was voluntary, that their responses would be kept confidential, and that they could withdraw from the study at any time without penalty. Participants were also requested to fulfill the questionnaire authentically and independently.

Given that participants were minors reporting NSSI and interparental violence, safeguarding procedures were implemented. After each survey wave, a life-education mental health course was arranged to help students recognize the importance of life. After the follow-up data collection, a risk assessment was conducted by research team members with training in clinical and counseling psychology, who evaluated the severity and urgency of any disclosed interparental violence exposure or NSSI. If the disclosure indicated serious risk, the student was referred to the school’s psychological counseling office, which then followed the school’s established crisis intervention protocol. Participants were also given contact information for the research team in case they experienced any discomfort or had questions about their rights as participants.

### 2.4. Data Analysis

SPSS 22.0 was used to perform preliminary analyses, including tests of data distribution, descriptive statistics, and correlational analysis. WFFV (skewness = 3.97, kurtosis = 18.12), WMFV (3.28, 12.53), T1 NSSI (2.22, 5.28), and T3 NSSI (2.30, 5.43) showed substantial skewness and kurtosis, exceeding the conventional cutoffs of Sk < |2.0| and Ku < |7.0| (Hancock et al., 2010), whereas ML-P (−0.22, −0.67) and ML-S (−0.28, −0.61) were approximately normally distributed. In addition, given the nested structure of the data (students within classrooms), we first computed intraclass correlations (ICCs) for the representative indicators of each latent construct to assess the degree of nesting. ICC values ranged from 0.007 to 0.092 across constructs, with search for meaning showing the highest ICCs (0.074–0.092) and the remaining constructs mostly below 0.06. Accordingly, all models were estimated using the TYPE=COMPLEX option in Mplus (version 8.3) with classroom as the cluster variable (38 clusters) to adjust standard errors for the nested data structure (B. O. Muthén & Satorra, 1995).

To address the potential impact of non-normality and clustering, all models were estimated using MLR, which provides standard errors and chi-square statistics robust to non-normality and non-independence (L. K. Muthén & Muthén, 1998–2017). Before examining gender moderation, we first estimated a single-group model in which gender was included as a control variable. In the multigroup model, gender served as the grouping variable instead and was therefore dropped as a covariate. To examine gender moderation, we first tested measurement invariance (configural, metric, and scalar) across gender using multigroup CFA (Putnick & Bornstein, 2016). Fit index changes were evaluated against recommended cutoffs: ΔCFI ≤ 0.010, ΔRMSEA ≤ 0.015, and ΔSRMR ≤ 0.030 (Chen, 2007; Cheung & Rensvold, 2002). TRd values were computed using the Satorra–Bentler correction (Satorra & Bentler, 2001). We then tested structural invariance by comparing unconstrained and constrained structural models. All models were estimated using MLR to obtain robust standard errors and chi-square statistics. Because MLR does not provide bootstrap confidence intervals, indirect effects were tested separately using ML estimation with 5000 bootstrap resamples, and bias-corrected 95% confidence intervals were computed (CINTERVAL(BCBOOTSTRAP)). The indirect effect was considered significant if the confidence interval excluded zero (Hayes, 2013).

## 3. Results

### 3.1. Preliminary Analysis

Common method bias (CMB) was preliminarily assessed using Harman’s single-factor test and an unmeasured latent method factor (ULMF) model. First, all 40 indicators were loaded onto a single factor. The single-factor model fit the data poorly (χ^2^(740) = 16180.889, *p* < 0.001, CFI = 0.322, TLI = 0.285, RMSEA = 0.118, 90% CI [0.117, 0.120], SRMR = 0.186). As a reference, the theoretically specified six-factor measurement model demonstrated good fit (χ^2^(725) = 1988.946, *p* < 0.001, CFI = 0.945, TLI = 0.940, RMSEA = 0.034, 90% CI [0.032, 0.036], SRMR = 0.036). The single-factor model fit substantially worse than the six-factor model, providing preliminary evidence that no single common factor accounted for most of the covariance among the indicators. Second, an unmeasured method factor was added to the six-factor measurement model. This model failed to yield an acceptable solution (χ^2^(692) = 2761.556, *p* < 0.001, CFI = 0.909, TLI = 0.898, RMSEA = 0.045, 90% CI [0.043, 0.047], SRMR = 0.516). The substantial deterioration in SRMR (0.036 to 0.516) and the decrease in CFI (0.945 to 0.909) indicate that the method factor could not be stably identified (Podsakoff et al., 2024; D. Tang & Wen, 2020). Taken together, these results provide preliminary evidence that CMB did not substantially distort the measurement structure, although neither approach can rule out common method bias entirely.

At T1, 20.20% of adolescents reported past exposure to WFFV and 26.00% reported past exposure to WMFV. For NSSI, 52.18% of adolescents reported engaging in at least one of seven NSSI behaviors in the past six months at T1; at T3, this proportion was 36.22%. The prevalence of specific behaviors at T1 was punching 36.22%, biting 30.42%, scratching skin 24.09%, inserting objects 20.62%, banging the head or body 20.50%, self-cutting 19.54%, and burning 10.10%. At T3, the corresponding figures were punching 26.57%, biting 24.71%, scratching skin 23.29%, inserting objects 19.56%, banging the head or body 18.66%, self-cutting 18.40%, and burning 15.18%. These figures indicate that the overall NSSI prevalence was driven largely by higher-frequency behaviors such as punching and biting, whereas more severe behaviors such as cutting and burning were considerably less common. Table 1 presents descriptive statistics and correlations. Both T1 WFFV and T1 WMFV were positively correlated with T1 and T3 NSSI, but negatively correlated with T2 ML-P and ML-S. T2 ML-P and ML-S were negatively correlated with T1 and T3 NSSI. Gender was positively correlated with both T2 ML-P and ML-S, indicating that girls reported higher levels of both. T1 SES was negatively correlated with T2 ML-S, indicating that higher socioeconomic status was associated with lower search for meaning at T2.

### 3.2. Testing the Mediating Effect of Adolescents’ Meaning in Life

Before testing the mediating effect of adolescents’ meaning in Life, we first evaluated the total effect (i.e., without meaning in Life as mediators) of interparental violence on NSSI after controlling for adolescents’ age, gender, SES, and baseline NSSI. The structural model demonstrated good fit to the data (χ^2^(478) = 1277.568, *p* < 0.001, CFI = 0.942, TLI = 0.936, RMSEA = 0.034, 90% CI [0.031, 0.036], SRMR = 0.036). WMFV and NSSI at T1 were positively associated with adolescents’ NSSI at T3 (βs = 0.08 and 0.36, *p*s < 0.05). WFFV was marginally significantly associated with adolescents’ NSSI at T3 (β = 0.09, *p* = 0.053). NSSI at T1 was positively associated with adolescents’ NSSI at T3 (β = 0.36, *p* < 0.001). Adolescents’ age, gender, and SES were not significantly associated with NSSI at Wave 3 in this model (β1 = −0.004, β2 = −0.006, β3 = 0.008, *p*s > 0.05). This model accounted for 18.6% of the variance in T3 NSSI.

**Figure 2:**
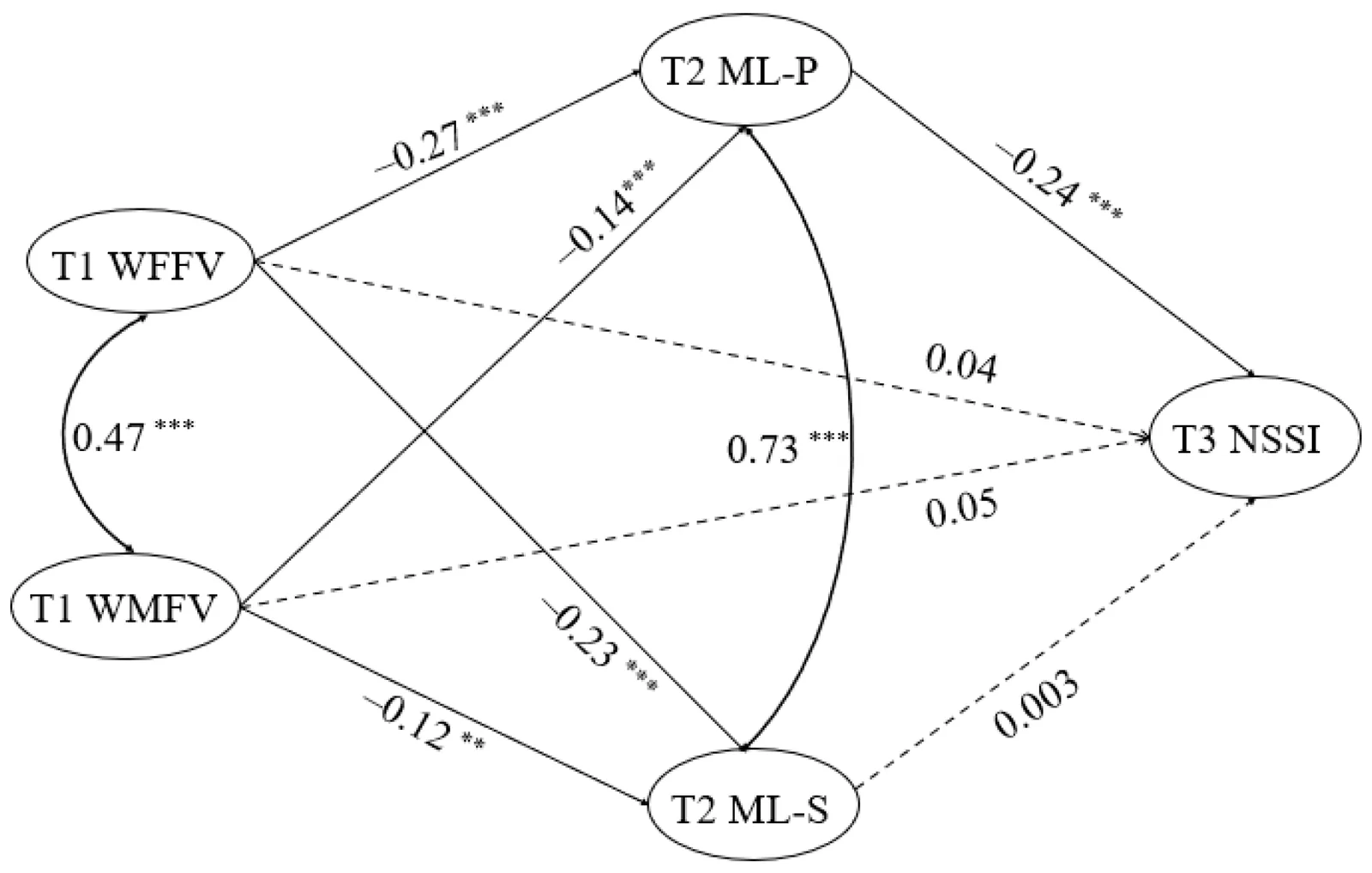
Standardized regression coefficients of the mediation model among WFFV, WMFV, ML-P, ML-S, and adolescents’ NSSI. Covariates and indicator variables are omitted for clarity. Dashed lines denote nonsignificant paths and the solid line represents a significant path. ** *p* < 0.01, *** *p* < 0.001.

A mediation model was then tested, with ML-P and ML-S as mediators, controlling for age, gender, and SES at both the mediator and outcome levels, and for T1 NSSI at the outcome level. The model demonstrated good fit, χ^2^(830) = 2212.199, *p* < 0.001, CFI = 0.944, TLI = 0.939, RMSEA = 0.033, 90% CI [0.032, 0.035], SRMR = 0.040. The results indicated that this model accounted for 13.2% of the variance in T2 ML-P, 9.7% of the variance in T2 ML-S, and 22.2% of the variance in T3 NSSI. As shown in Table 2 and Figure 2, more WFFV at T1 was associated with worse ML-P (β = −0.27, *p* < 0.001) and ML-S (β = −0.23, *p* < 0.001) at T2; more WMFV at T1 was linked to worse ML-P (β = −0.14, *p* < 0.001) and ML-S (β = −0.12, *p* = 0.001) at T2. Adolescents’ ML-P at T2 was negatively correlated with their NSSI at T3 (β = −0.24, *p* < 0.001). The association between T2 ML-S and T3 NSSI was not significant (β = 0.003, *p* = 0.952). Among the control variables, NSSI at T1 was positively associated with adolescents’ NSSI at T3 (β = 0.34, *p* < 0.001). In addition, gender was marginally positively associated with ML-P (β = 0.06, *p* = 0.053), and SES was marginally negatively associated with ML-S (β = −0.04, *p* = 0.059). No other control paths reached significance.

We then evaluated the mediating effects of ML-P and ML-S in the associations between T1 WFFV/WMFV and T3 NSSI. The results indicated that T2 adolescents’ ML-P not only mediated the relationship between T1 WFFV and T3 NSSI (indirect effect = 0.06, *p* < 0.001, bias-corrected 95% CI [0.035, 0.101]), but also mediated the relationship between T1 WMFV and T3 NSSI (indirect effect = 0.03, *p* = 0.001, bias-corrected 95% CI [0.016, 0.057]). In contrast, the mediating effects of T2 adolescents’ ML-S in these pathways were not significant (T1 WFFV→T2 ML-S→T3 NSSI indirect effect = −0.001, *p* = 0.952, bias-corrected 95% CI [−0.023, 0.022]; T1 WMFV→T2 ML-S→T3 NSSI indirect effect = 0.000, *p* = 0.953, bias-corrected 95% CI [−0.012, 0.012]). These findings revealed that both WFFV and WMFV were risk factors for adolescents’ ML-P and ML-S, but they were indirectly associated with adolescents’ NSSI only through ML-P.

### 3.3. Testing the Moderating Effect of Adolescents’ Gender

To examine whether gender moderated the associations among witnessing interparental violence, meaning in life, and NSSI, we first tested measurement invariance across gender and then tested structural invariance. As shown in Table 3, the configural invariance model showed acceptable fit, χ^2^(1434) = 2888.883, *p* < 0.001, CFI = 0.940, TLI = 0.934, RMSEA = 0.037, 90% CI [0.035, 0.039], SRMR = 0.042. The metric invariance model also showed acceptable fit, χ^2^(1468) = 3033.148, *p* < 0.001, CFI = 0.935, TLI = 0.931, RMSEA = 0.038, 90% CI [0.036, 0.040], SRMR = 0.049. The scalar invariance model showed acceptable fit, χ^2^(1502) = 3137.750, *p* < 0.001, CFI = 0.932, TLI = 0.930, RMSEA = 0.038, 90% CI [0.036, 0.040], SRMR = 0.048. As shown in Table 4, the TRd values were significant for both metric versus configural (TRd = 103.46, Δ*df* = 34, *p* < 0.001) and scalar versus metric (TRd = 133.63, Δ*df* = 34, *p* < 0.001). Although the TRd values were significant, this test is highly sensitive to sample size; changes in fit indices, which are less sample-size dependent, were all within recommended cut-offs (ΔCFI ≤ 0.005, ΔTLI ≤ 0.003, ΔRMSEA ≤ 0.001, ΔSRMR ≤ 0.007), supporting metric and scalar invariance.

Structural invariance was then tested to examine whether gender moderated the structural paths in the mediation model. The unconstrained structural model showed acceptable fit (χ^2^(1654) = 3400.617, *p* < 0.001, CFI = 0.931, TLI = 0.929, RMSEA = 0.038, 90% CI [0.036, 0.039], SRMR = 0.051). The constrained structural model also showed acceptable fit (χ^2^(1669) = 3431.061, *p* < 0.001, CFI = 0.931, TLI = 0.929, RMSEA = 0.038, 90% CI [0.036, 0.039], SRMR = 0.055). The TRd value was significant (TRd = 29.78, Δ*df* = 15, *p* = 0.013), but fit index changes were negligible (ΔCFI = 0.000, ΔTLI = 0.000, ΔRMSEA = 0.000, ΔSRMR = 0.004), likely reflecting the sensitivity of the chi-square difference test under a large sample rather than a substantive difference in structural paths. These results support structural invariance across gender, and no follow-up pairwise comparisons were conducted.

## 4. Discussion

Witnessing interparental violence has been consistently linked to adverse adolescent outcomes (e.g., Haj-Yahia & Basal, 2024; T. Lawrence, 2022), yet much of this work has focused on externalizing behaviors rather than NSSI. The present study aimed to contribute to this line of research by examining the longitudinal association between witnessing interparental violence and NSSI, with meaning in life as a mediator and gender as an exploratory moderator. We first examined whether T1 witnessing interparental violence was associated with T3 NSSI. After accounting for adolescents’ gender, age, SES, and baseline NSSI, both WFFV and WMFV at Time 1 were associated with NSSI at Time 3, although the association was significant only for WMFV and marginal for WFFV. When the two meaning-in-life dimensions were entered as mediators, only ML-P, and not ML-S, carried the indirect effect. These findings point to ML-P as one possible pathway linking witnessing interparental violence to NSSI. However, the exploratory analysis of gender moderation did not reveal significant gender differences in these associations.

### 4.1. Relationship Between Witnessing Interparental Violence and Adolescents’ NSSI

Previous studies have shown that exposure to interparental violence is associated with adolescents’ externalizing outcomes such as aggressive behavior (e.g., Gao et al., 2025; T. Lawrence, 2022). The present study extends this work by examining NSSI, a maladaptive behavior that is conceptually distinct from aggression and suicidal behavior. The present study found that T1 WMFV had a significant total effect on T3 NSSI, whereas T1 WFFV had a marginally significant total effect. In line with the functional model of NSSI, which conceptualizes it as a maladaptive strategy for regulating unbearable affect when other coping resources are insufficient (Nock & Prinstein, 2004), witnessing interparental violence may be associated with NSSI, although the specific affective mechanisms linking them were not directly measured in the present study. Notably, after accounting for the mediating role of adolescents’ sense of meaning in life, the direct effect of witnessing interparental violence on NSSI was no longer significant. This finding highlights the importance of considering the mediating role of meaning in life when interpreting the relationship between witnessing interparental violence and adolescents’ NSSI.

### 4.2. The Mediating Role of Adolescents’ Meaning in Life

Consistent with the stress process model (Pearlin et al., 1981), which posits that exposure to stressors depletes psychological resources and increases vulnerability to maladaptive outcomes, our findings suggest that witnessing interparental violence may deplete adolescents’ sense of meaning in life, which in turn increases their risk of NSSI. Specifically, when examining the mediating role of adolescents’ meaning in life in the relationship between witnessing interparental violence and NSSI, we found that ML-P, but not ML-S, mediated this association. Both WFFV and WMFV were negatively associated with adolescents’ ML-P and ML-S. An individual’s sense of meaning is shaped through relationships with the external world, with interparental relationships constituting a central component of this sense of meaning (Heine et al., 2006). When these relationships are characterized by violence, however, the relational foundation that supports adolescents’ sense of meaning may be compromised. These findings underscore the importance of a positive family climate in shaping adolescents’ sense of meaning in life and align with prior research indicating that unfavorable family environments are associated with lower perceived meaning in life (Shek et al., 2025; R. Zhang et al., 2017).

Although both WFFV and WMFV were negatively associated with adolescents’ ML-P and ML-S, they were indirectly associated with adolescents’ NSSI only through ML-P. This finding is consistent with Steger et al. (2008a), who found that searching for meaning does not necessarily lead to its presence and may be associated with greater distress such as dissatisfaction, anxiety, and depression, particularly when individuals experience a meaning-deficit state. A meta-analysis of 108 studies further supports this pattern, showing that search for meaning was positively correlated with psychological distress (He et al., 2023). More recent research has similarly indicated that ML-S does not necessarily protect against psychosocial problems (e.g., Onyekachi et al., 2024; Tian et al., 2024). In contrast, ML-P reflects an individual’s already perceived sense of purpose and meaning in life (Tian et al., 2024), which may serve as a protective factor against NSSI. This asymmetry is consistent with the theoretical distinction between presence of meaning as an established resource and search for meaning as an ongoing process (King & Hicks, 2021), which helps explain why only presence of meaning, and not search for meaning, mediated the association between witnessing interparental violence and NSSI.

Two further points about the mediation findings warrant attention. First, the latent correlation between ML-P and ML-S in our sample was substantially higher than typically observed in North American samples and closer to the pattern reported by Steger et al. (2008b) for Japan. This high correlation may partly account for why ML-S did not carry the indirect effect: when ML-P and ML-S overlap substantially, ML-S may contribute little unique variance beyond ML-P in this model, although cultural orientations were not directly measured in the present study. Second, these mediation findings should be interpreted considering their effect sizes. The standardized indirect effects ranged from 0.03 to 0.06, and adding ML-P and ML-S as mediators increased the explained variance in T3 NSSI from 18.6% to 22.2%, with most of that variance carried by baseline NSSI. These are small effects, consistent with meta-analytic findings on family variables as predictors of NSSI (Fox et al., 2015). Given the multiply determined nature of NSSI, the practical significance of the mediating role of ML-P should be interpreted with caution, and the findings should not be taken to imply that interventions targeting presence of meaning alone would substantially reduce NSSI risk.

### 4.3. Gender as a Moderator: An Exploratory Analysis

Regarding gender, the exploratory analysis did not find significant gender differences in the associations among witnessing interparental violence, meaning in life, and NSSI. This finding is consistent with several studies that have reported no gender differences in the effects of family violence exposure on adolescents’ psychosocial adjustment (e.g., Fagan & Wright, 2011; Gao et al., 2025). Given that the literature on gender differences in this area is mixed (e.g., T. I. Lawrence et al., 2023; Sokar et al., 2023) and that our gender moderation analysis was exploratory, this null finding should be interpreted with caution. It may indicate that the mediating role of meaning in life operates similarly for boys and girls, or it may reflect the limited statistical power to detect small moderation effects. Future research with larger samples and more diverse measures of gender-related constructs may help clarify whether gender moderates these associations.

### 4.4. Implications

Our findings have implications for understanding the consequences of witnessing interparental violence in adolescents. First, this study provides evidence that witnessing interparental violence is associated with NSSI. This expands current understanding of the consequences of family violence exposure, suggesting that witnessing interparental violence may be associated not only with externalizing outcomes but also with NSSI, a maladaptive behavior that is conceptually distinct from aggression. Given the small effect sizes, these findings should be viewed as preliminary. They suggest that child protection practitioners may consider prevention and intervention programs that target NSSI risk, but further research is needed to establish whether such programs are effective. Previous research indicates that programs focused on improving parental communication and conflict resolution, such as the Family Communication Project (FCP; Devonshire et al., 2022), may be effective in reducing adolescents’ exposure to interparental violence.

Second, this study simultaneously examined the mediating roles of ML-P and ML-S in the association between witnessing interparental violence and adolescents’ NSSI. The results indicated that only ML-P, and not ML-S, mediated this association. This finding highlights that ML-P and ML-S are interconnected yet distinct psychological resources for adolescents. Interventions aimed at preserving or enhancing ML-P in adolescents exposed to interparental violence may be a promising direction for future research. However, given the small effect sizes, interventions should not focus narrowly on presence of meaning as a primary target. Instead, a broader approach that addresses multiple risk factors, including family conflict and other known predictors of NSSI, may be more appropriate (Fox et al., 2015; Qu et al., 2023). Translating the present findings into intervention recommendations should therefore be done cautiously.

### 4.5. Limitations and Future Directions

Several limitations should be acknowledged. First, although this study employed a half-longitudinal mediation design, the findings remain essentially correlational and do not establish causality. In particular, the mediation model did not control for baseline meaning in life (T1 ML-P and T1 ML-S), so we cannot determine whether witnessing interparental violence is associated with changes in meaning in life over time or merely correlates with T2 levels. In addition, although we controlled for gender, age, SES, and T1 NSSI, residual confounding by unmeasured variables cannot be ruled out. These include direct child victimization, parental psychopathology, broader family dysfunction, adolescent depression or anxiety, family structure, and religious involvement, which is a potentially important source of meaning in life (Pfund et al., 2022; Skidmore et al., 2025) and may also be associated with lower NSSI risk. Future research should include baseline measures of the mediators, measure these additional variables, and use multi-source, multi-wave data collection to control for method variance more rigorously.

Second, the measurement of witnessing interparental violence and NSSI warrants caution. The WFFV scale showed high internal consistency alongside substantial floor effects, suggesting the items may be relatively redundant. The NSSI composite score averages frequency ratings across behaviors as different as cutting and biting, treating them as reflective indicators of a single latent tendency, which may not be the most appropriate conceptualization for behavior checklists; the internal consistency coefficients should therefore be interpreted with caution. We attempted to estimate censored and two-part specifications for NSSI, including models with Monte Carlo integration, but these models failed to converge, likely due to the complexity of combining a two-part specification with six latent factors, 40 indicators, and classroom-level clustering. We therefore retained the continuous specification. We also modified the original witnessing violence scale to focus specifically on interparental violence, which may limit comparability with studies using the original scale, and the measure did not include psychological or verbal aggression, potentially leading to an underestimation of the full impact. Future research should consider alternative operationalizations (e.g., a count of distinct behaviors, the highest frequency reported, or a two-part specification) and use measures that capture the full spectrum of interparental violence.

Third, the sample was drawn from only three public junior high schools in two provinces of China, and only students living with both parents were included. These sampling restrictions may limit generalizability to adolescents in other regions, school types, or family structures. In addition, participants were nested within classrooms; although we used TYPE=COMPLEX to adjust standard errors for clustering, some residual dependence within classrooms may remain. Future research should recruit samples from more diverse geographic regions and school types; include adolescents from single-parent, blended, and other family structures; and further examine classroom-level clustering.

Finally, although the present quantitative design allowed us to examine longitudinal associations among witnessing interparental violence, meaning in life, and NSSI, it cannot capture how adolescents subjectively interpret interparental violence or how they attempt to manage the resulting stress. Future research could benefit from qualitative approaches, particularly in-depth interviews with male and female adolescents, to examine how they encounter and interpret interparental violence and how they attempt to manage the stress associated with such exposure. NSSI may serve as one possible stress management strategy, and there may be gendered interpretations of parental violence and gendered motivations for self-harm.

## 5. Conclusions

Exposure to interparental violence is a widespread concern. The present study, using a three-wave longitudinal design, found that T1 WMFV had a significant total effect on T3 NSSI, whereas T1 WFFV had a marginally significant total effect. However, after ML-P and ML-S were included as mediators, these direct effects were no longer significant. Among the two meaning-in-life dimensions, only ML-P emerged as a small but statistically significant mediating pathway in this relationship. Contrary to the possibility that gender might moderate these associations, the exploratory multigroup analysis did not find significant gender differences in the structural paths, indicating that the mediating role of presence of meaning operated similarly for boys and girls. This study provides longitudinal evidence on the association between witnessing interparental violence and adolescents’ NSSI. It expands the literature on the link between exposure to family violence and adolescents’ NSSI and suggests that preventing interparental violence may be relevant for supporting adolescents’ sense of meaning in life and reducing their risk of NSSI. However, given the small effect sizes, future research should examine whether interventions targeting interparental violence and meaning in life can effectively reduce NSSI risk, ideally within a broader approach that addresses multiple risk factors.

## Figures and Tables

**Table 1:** Means, standard deviations, and correlations among the variables (*N* = 1489).

Variables	1	2	3	4	5	6	7	8
1. T1 WFFV	—							
2. T1 WMFV	0.41 ***	—						
3. T2 ML-P	−0.36 ***	−0.26 ***	—					
4. T2 ML-S	−0.29 ***	−0.22 ***	0.65 ***	—				
5. T1 NSSI	0.30 ***	0.18 ***	−0.23 ***	−0.17 ***	—			
6. T3 NSSI	0.24 ***	0.18 ***	−0.33 ***	−0.23 ***	0.38 ***	—		
7. T1 Age	0.03	0.01	−0.02	−0.01	−0.02	0.001	—	
8. Gender	−0.03	−0.03	0.07 *	0.06 *	−0.01	−0.01	0.01	—
9. T1 SES	−0.03	0.03	−0.05	−0.06 *	0.05	0.03	0.03	−0.02
*M* ± *SD*	1.17 ± 0.52	1.19 ± 0.45	4.82 ± 1.35	4.71 ± 1.55	1.34 ± 0.55	1.30 ± 0.55	13.13 ± 0.78	—

Note. Gender was coded as 0 = male, 1 = female. WFFV = witnessing father-to-mother violence, WMFV = witnessing mother-to-father violence, ML-P = presence of meaning, ML-S = Search for meaning, NSSI = non-suicidal self-injury. * *p* < 0.05, ** *p* < 0.01, *** *p* < 0.001.

**Table 2:** Unstandardized and standardized effects for the hypothesized mediation model (*N* = 1489).

Independent Variable	T3 NSSI				T2 ML-P				T2 ML-S			
	β	*p*	b	SE	β	*p*	b	SE	β	*p*	b	SE
Direct paths												
T1Age	−0.004	0.898	−0.002	0.019	0.003	0.897	0.001	0.009	−0.002	0.949	−0.003	0.047
Gender	0.007	0.803	0.008	0.033	0.059	0.053	0.035	0.018	0.051	0.154	0.145	0.102
T1SES	0.006	0.787	0.004	0.016	−0.013	0.598	−0.005	0.009	−0.041	0.059	−0.074	0.040
T1 WFFV	0.036	0.478	0.042	0.059	−0.269	<0.001	−0.169	0.057	−0.232	<0.001	−0.706	0.086
T1 WMFV	0.046	0.228	0.045	0.037	−0.142	<0.001	−0.075	0.023	−0.118	0.001	−0.301	0.094
T2 ML-P	−0.239	<0.001	−0.444	0.176								
T2 ML-S	0.003	0.952	0.001	0.018								
T1 NSSI	0.341	<0.001	0.315	0.048								
Indirect paths												
T1 WFFV (via T2 ML-P)	0.064	<0.001	0.075	0.019								
T1 WMFV (via T2 ML-P)	0.034	0.001	0.033	0.010								
T1 WFFV (via T2 ML-S)	−0.001	0.952	−0.001	0.013								
T1 WMFV (via T2 ML-S)	0.000	0.953	0.000	0.005								

Note. WFFV = witnessing father-to-mother violence, WMFV = witnessing mother-to-father violence, ML-P = presence of meaning, ML-S = Search for meaning, NSSI = non-suicidal self-injury. Residual covariances between WFFV and WMFV and between T2 ML-P and T2 ML-S were freely estimated.

**Table 3:** Model Fit Indices for Measurement and Structural Invariance Models Across Gender.

Model	χ^2^	*df*	*p*	CFI	TLI	RMSEA	RMSEA 90% CI	SRMR
Configural invariance model	2888.883	1434	<0.001	0.940	0.934	0.037	[0.035, 0.039]	0.042
Metric invariance model	3033.148	1468	<0.001	0.935	0.931	0.038	[0.036, 0.040]	0.049
Scalar invariance model	3137.750	1502	<0.001	0.932	0.930	0.038	[0.036, 0.040]	0.048
Unconstrained structural model	3400.617	1654	<0.001	0.931	0.929	0.038	[0.036, 0.039]	0.051
Constrained structural model	3431.061	1669	<0.001	0.931	0.929	0.038	[0.036, 0.039]	0.055

**Table 4:** Changes in Fit Indices for Model Comparisons.

Model Comparison	TRd	*p*	Δ*df*	ΔCFI	ΔTLI	ΔRMSEA	ΔSRMR
Model 1 vs. Model 2	103.46	<0.001	34	−0.005	−0.003	0.001	0.007
Model 2 vs. Model 3	133.63	<0.001	34	−0.003	−0.001	0	−0.001
Model 4 vs. Model 5	29.78	0.013	15	0	0	0	0.004

Note. Model 1 = configural invariance model; Model 2 = metric invariance model; Model 3 = scalar invariance model; Model 4 = unconstrained structural model; Model 5 = constrained structural model. TRd = Satorra–Bentler scaled chi-square difference test statistic. ΔCFI, ΔRMSEA, and ΔSRMR are computed relative to the less constrained model in each comparison.

## Data Availability

The data presented in this study are available on request from the corresponding author. The data are not publicly available due to privacy and ethical restrictions, as they contain sensitive information on adolescents’ non-suicidal self-injury and family violence, and the participants did not consent to public data sharing.

## Appendix A

**Table A1:** Model Comparison for the Dimensionality of the Witnessing Interparental Violence Scale.

Model	χ^2^	*df*	*p*	CFI	TLI	RMSEA [90% CI]	SRMR	ΔCFI	ΔTLI	ΔRMSEA	ΔSRMR
**WFFV**											
One-dimension (8 items)	125.998	20	<0.001	0.931	0.903	0.060 [0.050, 0.070]	0.036	—	—	—	—
Two-dimension (6 physical + 2 threat/property)	125.737	19	<0.001	0.930	0.897	0.061 [0.051, 0.072]	0.036	−0.001	−0.006	0.001	0.000
**WMFV**											
One-dimension (8 items)	56.479	20	<0.001	0.967	0.954	0.035 [0.024, 0.046]	0.033	—	—	—	—
Two-dimension (6 physical + 2 threat/property)	56.538	19	<0.001	0.966	0.951	0.036 [0.026, 0.048]	0.033	−0.001	−0.003	0.001	0.000

Note. WFFV = witnessing father-to-mother violence; WMFV = witnessing mother-to-father violence.

**Table A2:** Discriminant Validity of WFFV and WMFV.

Model	χ^2^	*df*	*p*	CFI	TLI	RMSEA [90% CI]	SRMR	ΔCFI	ΔTLI	ΔRMSEA	ΔSRMR
Two-factor (WFFV and WMFV correlated)	367.424	103	<0.001	0.935	0.925	0.042 [0.037, 0.046]	0.038	—	—	—	—
One-factor (all 16 items)	1478.978	104	<0.001	0.664	0.612	0.094 [0.090, 0.099]	0.180	−0.271	−0.313	0.052	0.142

Note. WFFV = witnessing father-to-mother violence; WMFV = witnessing mother-to-father violence.
